# “The biggest barrier was my own self”: the role of social comparison in non-traditional students’ journey to medicine

**DOI:** 10.1007/s40037-020-00580-6

**Published:** 2020-04-22

**Authors:** Rachel Ball, Kirsty Alexander, Jennifer Cleland

**Affiliations:** 1 School of Medicine, Medical Sciences and Nutrition, University of Aberdeen, Aberdeen, UK; 2 Research Department for Medical Education (RDME), UCL Medical School, University College London, London, UK; 3 Centre for Healthcare Education Research and Innovation (CHERI), Institute for Education in Medical and Dental Sciences, University of Aberdeen, Aberdeen, UK

**Keywords:** Widening access, Widening participation, Medical school, Application, Social comparison, Non-traditional

## Abstract

**Introduction:**

Social comparisons strongly influence an individual’s concept of self, their aspirations and decisions. This study investigates how non-traditional applicants used social comparison to shape their preferences, beliefs and predictions whilst preparing an application for medical school.

**Methods:**

Semi-structured interviews were conducted with 12 UK medical students from non-traditional backgrounds to explore their process of ‘getting ready’ for medical school, and the role social comparison played in their experiences. Thematic analysis was used to inductively develop themes in the data, before findings were interpreted through the ‘triadic model’ of social comparison.

**Results:**

Findings revealed that participants looked to the opinions of those with similar norms and backgrounds to accept their desire to study medicine. They sought the opinions of ‘experts’ to affirm a belief in their suitability but lacked confidence until success in crucial examinations ‘proved’, in their own view, that they had the ability to do medicine. Social comparison to peers who were perceived to be less committed to medicine, and to relatable role models, reassured participants that someone from their background could succeed in medicine.

**Discussion:**

Our findings further understanding about ‘how’ and ‘why’ exposure to relevant experts, peers and role models can positively influence application to medicine through the lens of social comparison. We recommend widening access initiatives promote and foster various opportunities for social comparison to help counter non-traditional students’ feelings of uncertainty about their ability and prospects, and to reorient their focus away from achieving the required grades before preparing the non-academic aspects of their application.

## Introduction

Medical schools in several countries across the globe are putting increasing focus on ‘widening access’. This initiative aims to attract a more diverse pool of applicants, with the goal of increasing social mobility and social accountability in the societies they serve [1–3].

Amongst UK children aged 11 years, aspiration to medicine is high irrespective of demographic factors [4]. However, at the point of application to medical school, this group of aspirants has become significantly more homogeneous: 75% of UK domiciled applicants have a parent in a professional or higher managerial position [5] and a large majority (80%) attend a minority (20%) of UK high schools [6].

To explain these disparities, studies have explored the additional challenges applicants from ‘non-traditional’ backgrounds may face when accessing the career. In the UK context, ‘non-traditional’ refers to those who live in areas of socioeconomic deprivation or low progression to university, attend low-attainment high schools, come from low income households, have been in state care or are entering medicine as a mature student, amongst other indicators [7]. The identified challenges include financial worries, perceived cultural barriers and lack of information [8–14]. Moreover, the odds of achieving the required academic entrance grades are steeply stacked against children in poorly resourced and low-attainment schools [10, 15]. Research suggests that a combination of multiple and intersecting factors—individual, social and cultural—need to come together to maintain aspiration to medicine over time [16–19].

The social and cultural factors related to aspiration to medicine for non-traditional students have been relatively well-researched, as have certain individual factors [20]. To the best of our knowledge, the individual factors which have been explored in relation to widening access to medicine relate to self-esteem [20, 21], self-efficacy [22, 23] and the personal preferences that drive motivation for medicine [19, 24, 25]. Our interest, on the other hand, is on the process of social comparison from the perspective of individual cognition, where literature and thus understanding is currently lacking.

The process of social comparison refers to individuals adapting their aspirations and levels of motivation according to perceptions of their own performance and attributes in relation to those of others [26–29]. Interpersonal comparisons are a core element of human experience and strongly influence an individual’s concept of self, decision-making and levels of aspiration [30]. Individuals evaluate through both social comparisons (e.g. praise given to peers), as well as comparing non-social measurements (e.g. test scores). Social comparison can be broadly defined as: “any process in which individuals relate their own characteristics to those of others” [31, p. 491].

Research has provided strong observational and experimental evidence to support the importance of social comparison in individuals’ evaluation-making, particularly when they feel uncertain and lack non-social information [32]. For example, Gore et al. highlight that relational factors, such as “perceiving oneself as ‘well above average’ relative to peers” is a significant predictor in relation to an interest in medicine [17, p. 227]. An exploration of the process of social comparison in shaping non-traditional individuals’ opinions towards medicine is therefore important: a better understanding of the role of social comparison on non-traditional individuals’ perceptions of medicine and their own ability to apply, may reveal important clues as to why some participants on widening access initiatives maintain an initial aspiration to study medicine, whereas others lose motivation and/or alter their goals.

To address this knowledge gap, we aimed to explore the effect of social comparison on non-traditional medical students’ aspirations and commitment to medicine. The following research questions guided our study:

1. How do medical students from non-traditional backgrounds experience the journey of getting ready for medical school?
2. What role did social comparison play in their experiences?

## Methods

This research is grounded in social constructivism. This approach draws on an idealist ontological assumption that there are multiple realities, each constructed as subjective meanings in the minds of individuals through engagement and sense-making of the world in which they live. The researcher’s aim is therefore to inductively interpret the meanings others construct about the situation or phenomenon under study as opposed to starting with a theory or pattern of meaning as would be undertaken by an objective worldview [33].

### Data collection

The study aimed to explore the experiences of non-traditional applicants to medicine who had successfully gained a place at a medical school in Scotland, UK. In the UK, the medical admissions process operates at a UK-wide level. Aspiring medics prepare an application form which details their academic achievements to date and predicted grades for any current studies, a supporting letter usually from a teacher, and a ‘personal statement’ which demonstrates their non-academic skills and achievements, as well as their motivation for medicine, typically through having completed extended work or voluntary experience in a relevant role. In this application, applicants may choose to apply to a maximum of four UK medical schools from 39 in total. The selection process then takes place at the level of the chosen medical schools and most commonly involves three components: high achievement in high school examinations if applying aged 17–18 years or having a suitable primary degree; performance on an aptitude test; and performance at interview to assess non-academic or personal qualities such as communication, motivation and integrity. The exact requirements differ by medical school [34]. Applicants can be rejected at each stage, for example, those who do not achieve the necessary examination outcomes do not progress to the later stages of selection.

The UK consists of four devolved nations (Scotland, Wales, Northern Ireland and Wales) which have differing educational systems. In Scotland, where this study took place, high school applicants sit their university entrance exams in their penultimate year of schooling when aged 16/17 and receive their results in August of the same year. If they apply and are offered a place on a medicine course, this offer may require them to achieve a smaller number of further qualifications in their final year at school. Individuals may also apply as graduates of a relevant undergraduate degree.

### Participant recruitment

Three of the four graduating medical schools in Scotland agreed to participate. Coordinators at participating medical schools emailed information about the study to students in all years. This invited students who had undertaken their pre-university schooling anywhere in the UK and who self-identified as from a non-traditional background and/or had been eligible to attend university widening access activities to volunteer to participate. We purposively employed broad eligibility criteria to encourage students from a range of groups and backgrounds to participate—e.g. from those who attended poor performing high schools, to mature or disabled students. Interested students were requested to contact the research team directly for more information and to arrange an interview.

### Interviews

Semi-structured interviews were conducted at the participants’ medical schools by a research assistant supervised by one of the authors (JC). Interview questions were developed on the basis of existing literature on non-traditional students’ experiences of getting into medical school and being a medical student. Participants were encouraged to discuss: when and why they decided to apply to medical school; barriers during the application process; sources of support; experiences of widening access initiatives; any perceived feelings of ‘difference’ between themselves and their peers both at secondary and medical school; and their plans for the future. Prompts were used to explore responses in more depth where required, and new topics raised in early interviews were included as open questions in later interviews.

### Data management and analysis

Interviews were transcribed verbatim, corrected for accuracy, anonymized and uploaded into NVivo 11 (QSR International Pty Ltd, Doncaster, Vic, Australia). We initially conducted a primary-level thematic analysis of the data following steps outlined in Braun and Clarke [35]. RB undertook the process of preliminary coding to look for themes associated with the research questions, with guidance from KA and JC to ensure the distinctiveness of the codes. RB discussed and reviewed codes with KA and JC at regular intervals to assist with recognizing and developing themes and to consider any researcher influence upon analysis. Subsequent cycles of coding and theme generation were led by RB, with KA and JC contributing to code confirmation, monitoring the trustworthiness of the process and offering critical discussion of emerging findings [36]. Any coding disagreements were addressed via team discussion.

Following this inductive and iterative data analysis, the research team were struck by a reoccurring integrative theme of ‘comparison’ permeating strongly across several distinct theme clusters. Given this, and in order to draw out this theme for full exploration, we investigated possible conceptual frameworks to ‘illuminate and magnify’ [37] the process of social comparison within the identified themes. Conceptual frameworks are developed from well-established theories or models and concentrate researcher focus on a specific aspect of a problem or topic, to offer an enhanced understanding from one perspective [38, 39].

### Conceptual framework

Social comparison theory stems from Festinger’s seminal work published in 1954 which posits that we compare ourselves with others in order to evaluate or to enhance some aspects of the self [40]. Social comparison theory has evolved over time, now showing that individuals are active, subjective evaluators driven by a wide range of motives, thus aligning with a social constructivist approach [31, 32]. We adopted Suls, Martin and Wheeler’s triadic model of social comparison [41] to interpret how our participants evaluated their opinions of applying to medicine in relation to their social experiences. This model incorporates three distinct types of opinion comparison:

1. To help individuals assess their preferences (e.g. to ask: “do I like X?”)
2. To test an individual’s beliefs (e.g. to ask: “is X correct?”)
3. To inform predictions (e.g. to ask: “will I like X?”)

Each type of opinion—preferences, beliefs or predictions—has a different comparison dynamic and therefore individuals look for three different sets of references to provide the highest potential for a meaningful and accurate comparison. Each of these aspects is illustrated further with examples from our findings in the Results section.

### Reflexivity

Qualitative data analysis is influenced by the researchers’ worldview and the context in which the research is performed [42]. At the time of the study RB was an undergraduate medical student; KA was a PhD candidate with a professional background in designing and implementing widening access initiatives; and JC is a psychologist and experienced medical education researcher. The team met at regular intervals to explore decisions taken during analysis and to challenge developing interpretations from a range of perspectives.

### Ethical considerations

Permission to conduct this study was granted by the College Ethics Review Board for the College of Life Sciences and Medicine at the University of Aberdeen: CERB/2013/7/920. Ethical approval was granted and institutional consent obtained prior to data collection. All procedures performed were in accordance with the ethical standards of the institutional and/or national research committee and with the 1964 Helsinki Declaration and its later amendments or comparable ethical standards. All participants received verbal and written information in advance of the study, as well as assurances of confidentiality, anonymity and data security. All provided written consent before participating.

## Results

In this section, firstly we describe the participants. We then explore the role social comparison played in participants’ experiences through interpreting these using each aspect of the triadic model described earlier [41]. To preserve their anonymity, participants and their medical school are referred to with a randomly assigned number and letter: e.g. Student 2, Medical School A.

### Descriptive results

Twelve medical students from three Scottish medical schools took part in the study: five from Medical School A; four at Medical School B; and three at Medical School C. All had entered on standard-entry routes (i.e. not through pre-medical widening access or accelerated graduate routes) and were in various stages of their medical degree course.

Participants were from the UK and self-identified as from non-traditional backgrounds through their involvement in, or eligibility for, widening access initiatives. Ten participants had entered medical school either directly from high school or after a gap year. The other two participants had completed at least one university-level degree before starting medicine, having chosen not to apply to medical school directly from high school for reasons explored below. Participants had grown up in a mix of rural and urban areas. This diversity of experiences, in combination with the complex range of social, cultural and individual factors influencing the participants’ choices, meant that we were careful to preserve the individuality of participants’ narratives during analysis, as well as explore any similarities.

Interviews lasted an average of 53 min, range: 27–87 min. Two students were interviewed together at their request: students 7 and 9 at Medical School A.

### Assessing preferences

Within our model of social comparison theory, to assess preferences individuals prioritize the judgements of those with similar attributes (e.g. tastes, abilities or background) to assess whether their own opinion is personally appropriate. Comparison can thus act as social validation and/or acceptance of an opinion.

In this study, participants reported an interest in medicine from a young age and described how they saw medicine as an elite and aspirational profession. However, they experienced doubts about their academic ability and thus were uncertain about whether aspiring to medicine was appropriate for them:

*For a long time, [medicine] had been the best. The standards for getting in were so high. The qualities that we were looking at, were qualities I very much admired—Student 4, Medical School C*

*I didn’t think I was the sort of person that could study medicine, like, smart enough… I didn’t think that it was a realistic dream—Student 3, Medical School C*

To address this uncertainty and assess their preference for medicine, participants reported seeking the opinions of families, peers and teachers. Participants found parents and friends supportive, accepting and validating their aspiration to medicine:

*My parents were really supportive. They don’t have a medical background at all, but they were just supportive in that anything that I wanted to do they would try and support me with it, just moral support or financial support, whatever they could afford.—Student 5, Medical School B*

*My friends also helped me a lot through that and they told me: “no, don’t think you’re not good enough. Come on, keep applying if that’s what you want to do”—Student 11, Medical School C*

Although aspiring to medicine was not a norm of their wider social group, the validation of parents and friends helped participants feel comfortable with, and more committed to, their preference. Although some participants also found their teachers supportive, others reported that teachers were less likely to accept or validate an aspiration to medicine in someone of their background:

*If you’re the son of a lone parent your first stop is in teaching and then your children go into medicine was always the thought process. It was just very much maybe go into PE teaching, you were interested in sport. Medicine was just a crazy idea—Student 10, Medical School B*

Notably, all participants who were eventually successful at securing a place in medicine had at least one source of strong acceptance and support of their choice to consider medicine.

### Assessing beliefs

When testing beliefs, individuals use social comparisons to evaluate whether their opinion is valid or correct. Those with recognized experience or status regarding the topic are considered experts, and are most influential during comparison, particularly if individuals perceive these experts to hold similar base values in common with their own. To test their belief that they could be suitable for medicine, participants looked to teachers, exam results and other applicants.

As mentioned above, our participants lacked confidence in their academic ability, and this undermined their belief in their ability to apply/do medicine. To evaluate this opinion, participants looked to their teachers, who could be considered experts on academic ability and potential. However, although some participants found teachers supportive, others felt they gave them the impression that they would ‘never make it’ and thus left them feeling uncertain:

*People around me didn’t want me to get my hopes up as well. So, it was, what about nursing? What about occupational therapy?—Student 3, Medical School C*

This negative reinforcement led many to consider alternative, more feasible career paths, such as teaching, science, and other allied health professions. Yet, although teachers’ views did thus have an effect, participants were not completely deterred, possibly because they did not see teachers as sufficiently ‘expert’ on identifying academic ability or potential for medicine. More influential than teachers was the experience of achieving the required grades. Participants described this as the major trigger that solidified their belief in their suitability for medicine and catalysed them into preparing an application:

*I think for me the biggest barrier was my own self, but I think, once I got my [high school] exam results, I thought, I can do this now. And, once I was sure that I could do it, then I don’t think much could have really stopped me.—Student 3, Medical School C*

This strong affirmation of belief is consistent with social comparison theory which posits that self-evaluation via non-social information, for example results in standardized tests, termed ‘objective’ evidence by Suls, Martin and Wheeler [32, 41] is prioritized over evaluations made via social comparison.

Although they were now more confident that they had the academic ability to apply for medicine, participants felt ‘lost’ as to how they fitted other, non-academic selection criteria. To address this, they sought out contact with advisors who were perceived as experts—people in, or associated to, the medical profession, for example, qualified doctors, medical students and admissions staff—through social networks, university outreach groups and online forums:

*When you come from a poorer background you don’t have the network of people who can advise you. You don’t know any doctors. You don’t know anybody who’s actually maybe gone to university before other than your teachers at school, so it’s very hard to arrange all of these extras that you need to get into medical school—Student 10, Medical School B*

With highly expert sources largely unavailable or hard to reach, participants looked to others they perceived as having more knowledge or experience, and with common goals—other applicants. Unfortunately, for many participants, this process highlighted the weaknesses in their own preparation and left them feeling disadvantaged in comparison to those who trusted in their ability to do medicine, and had thus been preparing their application for substantially longer:

*[I had to] build up a portfolio and my personal statement from that point onwards which was quite difficult because I’d obviously got a short period of time to get it done whereas [in comparison to] people who knew from first year secondary school they wanted to do it [apply to medical school]—Student 8, Medical School A*

*The pure breeds—I have to admit, I am kind of envious of some of their motivation, they’re born and bred for medicine—Student 7, Medical School A*

A unifying theme in the participants’ decision-making about whether or not they would be suitable for medicine was deep uncertainty. Student 7’s use of the term “pure breeds” positions him in contrast to this other group, who were implied to be predestined for medicine, possessing a motivation that was not accessible to non-traditional students. Participants’ uncertainty seemed to have been prolonged by the lack of access to others who could be perceived as sufficiently expert to assess both their academic and non-academic suitability.

### Informing predictions

According to the triadic model, when individuals are uncertain about whether they will enjoy a future situation, how they will react or what the consequences might be for them, they may test out their predictions through a comparison to the reactions of others who: a) have already experienced the activity/situation; *and *b) consistently share relevant attributes and/or similar opinions with the individual.

Participants viewed interactions with medical students or doctors from similar backgrounds, or who had faced similar setbacks and challenges, as particularly relevant in this respect:

*He [the doctor] was like: “well, I grew up in a tenant flat in Glasgow. I had absolutely nothing and I went and did medicine.” And he said, you know: “I gave you this opportunity to do this week [of work experience] with me, because I was in that situation. I didn’t know anything, I was the first medic in my family”. And it was so nice to hear from him, because he’s now… quite a well-known consultant, in radiology, and… he’d come from the same sort of background as me—Student 9, Medical School A*

Half of our participants were initially unsuccessful in their applications. This rejection might have been seen as a strong non-social prediction of unsuitability for the profession. However, our participants reflected on their experiences and comparisons and concluded that the problem was not their unsuitability but rather their lack of preparation for the application:

*I remember asking if I’d given it my best that year, or had I done what I thought I needed to do, rather than everything I could do, and I thought back and I thought, well, I probably did what I needed to do and I thought, well, what’s my best? I’ll give it my best and if it doesn’t work out this time, that’s fine—Student 4, Medical School C*

Their motivation to improve their application and reapply was notably boosted when they compared their actions with those of peers whom they had seen reapply without sufficient effort or reflection:

*I would never just send off the same application again I was always constantly looking at well what could I do that would be better the next time. I’ve seen people applying as well, almost half-heartedly, and sending off an almost identical application [for a second time] and then be disappointed when they’ve not got in. Not being quite so brutal as to say: “well what did you expect? You haven’t changed anything”—Student 1, Medical School A*

Another comparison group was that of the “*pure breeds*” (Student 7, full quote above) whose backgrounds had provided them with more certainty about their path to medicine and who had subsequently been successful at achieving a place in medicine. Although our participants generally saw themselves as disadvantaged in comparison with this group, they also identified comparative strengths:

*I think that they all were spoon fed, like with their work, but we’re used to working hard, but more on our own, and independent—Student 7, Medical School A*

*My communication skills and maturity developed [during my year out] because I was in three or four different jobs, meeting new people. A professional standard was expected of me. I think it really benefited me, especially when I first came to university—Student 3, Medical School C*

Finally receiving a place in medicine was a substantial confirmation of their prediction that altering their applications would be key, and that they would succeed within the career:

*Being able to cope with the application process… has given me confidence that I’ll be able to cope with [being] a doctor—Student 9, Medical School A*

## Discussion

This study investigated the processes of social comparison amongst medical students from non-traditional backgrounds as they considered and prepared an application to medical school. Through our analysis, we aimed to provide a nuanced and novel understanding of the influence of social comparison on aspiration for medicine during the process of preparing to apply, and thus help explain how and why this has emerged as a significant predictor of an interest in the profession in other work [17]. Findings, interpreted through the triadic model [41], revealed the ways in which participants compared others’ opinions, behaviours and achievements to their own when they were uncertain about: whether their aspiration for medicine was appropriate; whether they possessed the academic and non-academic ability to apply to medicine; and whether they would be successful in their application and career. Social comparisons were frequent and heavily referenced in participants’ stories of their experiences. By questioning the purpose of these comparisons, the participants’ choices of comparator and their reported effect, we were able to better understand this complex, prevalent and influential process.

Findings revealed, however, that participants struggled to resolve uncertainty about whether or not an aspiration to medicine was appropriate for them using social comparisons alone. Instead, only success in examinations solidified their belief in their academic abilities, and this then acted as the catalyst for action in terms of preparing an application to medical school. This finding corroborates a key understanding in the literature: that uncertainty about their own academic abilities is a major barrier discouraging non-traditional pupils from considering medicine [6, 16, 20, 43]. It is also consistent with social comparison theory, which understands self-evaluation via non-social, termed “objective”, information (e.g. results in standardized tests) to be prioritized over evaluations made via social comparison [32, 40]. However, this study adds novel understanding of the impact of these aspects: participants reflected that their need for non-social reassurance through achieving the grades before taking further action in preparing the non-academic aspects of their medical applications was a substantial disadvantage, as it left them very little time to gain the necessary non-academic skills and experiences for their medical school application.

We suggest this focus on getting the grades is reasonable given that academic success is enshrined as the first hurdle in the selection process. However, it is also problematic as it reinforces the belief that academic ability is the most important indicator of suitability for medicine, deemphasizing the need for appropriate personal qualities, skills and motivations. This tension exists elsewhere, as admissions deans wrestle with how best to assess potential for medicine [44].

Findings also show that participants seemed to solidify their commitment to medicine primarily through both downward comparison to peers, who they perceived to be less motivated and only much later on, upwardly, to other applicants who they perceived as better prepared. The influence of downward comparisons to those less fortunate and upward comparisons to higher achievers is debated, and modern studies appear to conclude upward comparison is more pervasive and positive in the classroom [27, 29, 30, 45]. We suggest that context is important: our participants reported few, or no, peers to whom they could upwardly compare themselves. This is an inherent issue in widening access to medicine—if a pupil is the only person in their year group who has the notion to apply for medicine, then they have no immediate comparators. Moreover, if they are at a school with lower performance outcomes, then pupils from the school who do apply for medicine are less likely to achieve the necessary grades and thus more likely to be unsuccessful—again meaning no upward comparison opportunities for others.

Finally, our study further reinforces the well-known importance of supportive family and friend groups in validating, and thus supporting, non-traditional applicants to aspire to medicine and of role models in helping individuals from non-traditional backgrounds commit to the profession [16, 19, 20, 43, 46, 47]. Our study, however, helps explain why role models are most effective if they are relatable, through the mechanics of social comparison [41, 46, 48].

Students with similar demographics, preferences and career intentions tend to cluster at different Scottish medical schools [49] and therefore this study was strengthened by its multi-centre approach. Participants were diverse, and included a mix of genders, came from both rural and urban areas of the UK, and had taken varied paths into medical school (directly from school, after a gap year, or as graduates). We described our participants and context in detail so other researchers can evaluate the extent to which our data and conclusions are transferable to their context.

It important to consider that our participants do not represent all non-traditional medical school applicants. Half the participants were required to reapply at least once, which suggests that they may be part of a specific subgroup both demographically [50], but also in terms of their growth mindset [51]—these applicants seemed to possess the grit to draw determination from initial failure rather than discouragement [52]. Moreover, all our participants were successful in their medical applications and were progressing through the standard medical courses at their respective schools. These participants are therefore perhaps unusual in their willingness and ability to preserve and adapt to the demands of medical application and study. A larger participant group would have allowed a yet wider range of views and experiences to have been included and boosted credibility. However, as we aimed to explore in-depth the complex process of social comparison within participants’ experiences, a small group of interviewees was appropriate [53, 54]. To combat bias towards a particular viewpoint, we made a conscious effort during analysis to give equal weight to each participant’s account in the analysis and write up, despite differences in experiences and levels of eloquence. Nonetheless, with such small numbers, some participants’ stories may have influenced interpretations more strongly and are quoted more frequently. Numbers of non-traditional students at Scottish universities, although slowly increasing, remain low overall, giving a small group of potential participants [5].

At the time of analysis RB was a medical student, which may have also had implications on the analytic process: for example, RB may have been more likely to identify themes that resonated with her own experiences of application. Measures such as the creation of a reflexive statement and critical discussions with team members were taken to minimize any potential bias.

Our participants self-identified and volunteered as non-traditional students. Other students with the same backgrounds may not have come forward as they did not identify themselves as such or may not have wished to be linked with this term. Two participants wished to be interviewed together. This group interview may have influenced their willingness to share their personal experiences and opinions honestly, due to concerns over acceptability to the other. On the other hand, as they listened, clarified, discussed and reflected upon the other’s contributions they, as individuals, may have offered a more considered, deeper response and contribution to the discussion [55].

The transferability and usefulness of our findings are enhanced by the clear use of social comparison theory [38], within the structured framework of the triadic model [41]. Future studies could use this model to further our understanding of social comparison within different groups. For example, we could ask whether medical students from traditional backgrounds use social comparison in similar ways, and whether they can access opportunities for comparison that are currently unavailable to those from non-traditional backgrounds. Social comparisons may also be more influential for the current and subsequent generations of medical applicants, as social media and online communications and information may increasingly facilitate and emphasize comparisons. Studies investigating any changes to the perceived importance, and changing role, of social comparison over time could thus also provide interesting results.

The findings of this study open up some interesting questions for widening access initiatives. UK universities receive dedicated governmental funding to run these initiatives to help meet governmental expectations of wider participation. High-quality preparation for a UK medical school application is time-consuming [6], and our participants felt disadvantaged if they had waited to prepare their applications until after they received their exam results (results are released in August, applications close mid-October). This might be particularly a concern in countries with similarly onerous requirements: as described earlier, in the UK, in addition to the very high academic grade requirements, applicants are required to sit up to three aptitude tests, undertake an extended period of work or voluntary experience (not necessarily clinical), complete a written online application process and prepare thoroughly for interviews. In addition, each of the 39 UK medical schools has differing selection criteria and attributes, and applicants may only initially apply to four: this requires substantial research and knowledge of the system to make strategic choices. Even the graduate participants who had experience of applying to university reported looking to others for reassurance and advice during their medical application, which entails a different and more involved process. This suggests widening access initiatives should consider increased support for individuals at this very late stage, perhaps straight after exam results are announced, for example a preparation boot camp. The UK Medical Schools Council’s booklet [34], which collates the requirements for all medical schools in a standardized format, is a useful addition for example. The timing of applications and results may be different in other contexts, but the finding that time pressure can be an issue for applicants from non-traditional backgrounds merits further exploration.

Concurrently, students who aspire to medicine should be encouraged to start preparing their non-academic skills and applications earlier and, importantly, to see the value in doing so, despite competing in a system which still reinforces academic achievement as the primary hurdle to be overcome. Our findings also suggest that pupils may require stronger social validation of their aspiration, perhaps from trusted experts or greater exposure to opportunities for social comparison, for example through widening access activities. Earlier and stronger reinforcement of non-social/objective feedback on their academic progress in itself may be problematic, due to the influence of students’ own self-esteem and views on ability during the feedback process [51, 56]. Additionally, this may work to reinforce the impression of academic achievement in high school as paramount within a medical application, over academic ability in complement with desirable non-cognitive skills and personal qualities. Gateway or extended access routes [34] should be more widely promoted to reassure potential applicants that alternative routes into medicine do exist should they not meet the standard-entry grade requirement.

In conclusion, this study reveals three important processes of social comparison at work as UK non-traditional individuals consider, and prepare for, an application to medical school. Findings illustrate the importance of exposure to relevant experts, peers and role models and thus recommends widening access initiatives promote and foster various opportunities for social comparison.
